# How Does Age Affect Injury Characteristics in Young Elite Footballers?—A Prospective Cohort Study of a German Youth Academy

**DOI:** 10.3390/jcm12216938

**Published:** 2023-11-05

**Authors:** Johannes Weishorn, Ayham Jaber, Raphael Trefzer, Severin Zietzschmann, Ralph Kern, Jan Spielmann, Tobias Renkawitz, Yannic Bangert

**Affiliations:** 1 Department of Orthopaedics, Heidelberg University Hospital, Schlierbacher Landstrasse 200a, 69118 Heidelberg, Germany; 2 Ethianum Heidelberg, Fehrentzstrasse 2, 69115 Heidelberg, Germany; 3 TSG 1899 Hoffenheim Fußball-Spielbetriebs GmbH, Horrenberger Straße 58, 74939 Zuzenhausen, Germany; 4 TSG ResearchLab gGmbH, Horrenberger Straße 58, 74939 Zuzenhausen, Germany

**Keywords:** adolescent, growth plate, muscle injury, age group, injury burden, epidemiology, paediatrics, soccer, football, sports medicine

## Abstract

Background: Little is known about age-related changes in injury characteristics and burden, and existing data are inconsistent, highlighting the need for new studies on this topic. This study aimed to describe age-related injury risk, severity and burden in a German elite youth football academy. Methods: A prospective cohort study was conducted in the 2012/2013 season, reporting 109 time-loss injuries among 138 young athletes playing at an elite football academy in Germany. For the most severe injuries, the injury burden in the different age groups was considered separately. Results: Athletes missed a total of 2536 days of exposure, resulting in an overall incidence of 2.6 per 1000 h (1.7–3.0; 95% CI) and a burden of 60.6 days lost per 1000 h (40.8–80.3; 95% CI). The incidence and burden of joint sprains and muscle injuries were higher in the older age groups. Physeal injuries peaked in the U14 age group during the pubertal growth spurt. Bone injuries and contusions showed no age trend. Conclusion: Injury characteristics vary with age. The overall incidence, severity and burden of injuries increased with the age of the athletes. To ensure the optimal development of young athletes, it is important to be aware of the differences in injury susceptibility between age groups in order to implement tailored prevention programmes.

## 1. Introduction

Injuries in youth football (soccer) lead to limited participation in training and competition, making injury prevention a key element in improving the development and performance of young athletes [1]. To achieve this goal, a thorough understanding of injury characteristics is important in order to identify potential causes of injury and to take preventive interventions [2]. The lack of knowledge about effective and safe training management of adolescent athletes contributes to their unnecessary exposure to high risk of injury [3].

The general patterns of injury in elite male youth football players and their adult counterparts appear to be consistent, especially in older athletes, but age-specific trends during the developmental process are poorly described [1,4]. The body of young athletes undergoes an individual dynamic process during puberty, characterised by vulnerable structures and periods of rapid growth [5,6]. This is one proposed explanation for the increased rates of growth-related injuries and the greater injury burden observed during the period when height and weight typically change the most [7,8,9,10,11,12]. Reduced bone mineral density, neuromuscular deficits, increased tension on muscle attachments, and increased training and match loads in youth football academies are also discussed in this context [13,14].

Due to methodological inconsistencies in published studies of injury in elite youth football, there is a wide variation in reported injury outcomes. The distinction between “per player season” and “per 1000 h of exposure” is an issue in the reporting of injury data. The specification per player season only suggests that exposure is taken into account when reporting incidence and burden. However, as exposure during a season varies between teams, leagues and countries, the specification per 1000 h of exposure is concrete.

Two recent studies from English football academies reported an injury rate of 0.4 to 1.3 per player season [7,8]. However, these studies do not report player exposure, so an estimate of the injury risk (incidence per 1000 h of exposure) is not available. Other studies report an injury risk between 2.6 and 12.0 per 1000 h of exposure [15,16]. Furthermore, the focus of published studies is often limited to reporting injury rates without considering the impact of individual injuries on participation in training and play. In this context, it is also important to understand the burden of these injuries [17,18]. In particular, by considering the severity of individual injuries, it is possible to distinguish between common but minor injuries (e.g., contusions) and rare but severe injuries (e.g., fractures), providing a more nuanced understanding of the overall impact of injury on young athletes’ participation in training, play and therefore their development [17].

The limited amount of information from a few different cohorts and age groups, with inconsistent data, highlights the need for new studies on this topic. The aim of this study was to describe the age-related injury risk, injury severity and injury burden in relation to the injury types observed in a German elite youth football academy.

## 2. Materials and Methods

The present study examines prospectively collected injury data from a cohort of 138 young male athletes who played in the U12–U19 groups of an elite youth academy in Germany during the 2012/13 season. Inclusion criteria were defined as club membership throughout the study period, complete injury registration during the study period, and complete registration of training and match exposure during the season of data collection. The study period covered one full season, starting with the pre-season in July 2012 and ending at the end of May 2013. The Strengthening the Reporting of Observational Studies in Epidemiology (STROBE) statement was used for appropriate reporting. of this study [19].

The academy follows a structured training programme that gradually incorporates both athletic and football-specific training. The frequency of training varied by age group, ranging from an average of 3 sessions per week for U12 and U13 to an average of 5 sessions per week for U16, U17 and U18. Match participation ranged from 44 matches for U16 to 74 for U13, with match duration ranging from 60 min (U12 and U13) to 90 min (U19). U19 players had the highest match exposure, accumulating 891 h. Additional training or matches related to junior national teams were not included in this study. The participants were not involved in the design or interpretation of the study.

### 2.1. Injury Monitoring

In the event of an injury, all players were required to contact the club’s medical staff. The doctor or physiotherapist carried out a physical examination and, if necessary, ordered the appropriate diagnostic tests to classify the injury. In all cases, the affected body region (location), type of injury (diagnosis) and nature (acute/overuse) were recorded and documented in a spreadsheet database according to the methodology described in the consensus procedure by Fuller et al. [20]. The exact mechanism of trauma was not determined.

The diagnosis was made after an interprofessional exchange between physiotherapists and physicians, in consensus with the players and coaches and in full awareness of the situation. The dates of injury and return to sport, defined as the time of full return to training or match play, were documented. Injuries were followed until the end of the rehabilitation protocol. The exact date of return was estimated by the treating clinician at the end of the observation period in the case of persistent injuries [20]. Injury severity was divided into three categories and classified as mild (4–7 days lost), moderate (7–28 days lost) and severe (>28 days lost). Minor injuries lasting <3 days were not recorded.

Injury records were checked weekly for completeness. Non-sport-related injuries, such as influenza infections or general illnesses, were excluded from this study.

### 2.2. Exposure Monitoring

Exposure data for the U17 and U19 teams were recorded electronically by their respective coaches. In contrast, exposure data for the U12 to U16 age groups were documented by the team physician and medical staff. Match load was monitored on an individual basis, while training load was assessed at team level.

### 2.3. Data Analysis

For descriptive analysis, parametric data were reported as mean and standard deviation, while skewed data were reported as median and interquartile range. Frequencies were presented as both absolute numbers and relative percentages to facilitate comparisons in terms of location, type and cause of injury. Significance was assessed at a *p*-value of less than 0.05. Where possible, results are presented visually using graphs. SPSS 26.0 (IBM, Armonk, NY, USA) and Microsoft Excel version 2308 (Microsoft, Redmond, WA, USA) were used for statistical analysis and graphical presentation.

The primary outcome of age-dependent injury incidence was determined by calculating the number of injuries per 1000 h of exposure in the different age groups and reported both overall and grouped by injury type [21]. Due to the skewed distribution, the median injury severity was used to calculate the age- and pathology-specific injury burden as the second main outcome measure, as the product of the number of injuries and the injury severity [21]. The injury burden was expressed as the number of days lost per 1000 h of play and the dispersion was expressed as the 95% CI assuming a Poisson distribution [21]. Detailed operational definitions used throughout the study are presented in Table 1.

## 3. Results

A total of 138 players with recorded training or match exposure were screened for eligibility. None were excluded, and all played at least part of a season. Mean age at start of season was 14.9 years with SD of 2.1 years. Cumulative exposure was 36,638 h of training and 5335 h of match play with a mean season length of 40 weeks (range 35–45 weeks).

### 3.1. Main Injury Outcomes

A total of 109 time-loss injuries were recorded. On average, each injury prevented players from participating in training and match-related activities for approximately 23 days, with an average of 0.8 injuries per player season. The overall incidence was 2.6 per 1000 h (1.7–3.0; 95% CI). The age-related incidence ranged from 1.1 in U13s to 4.0 in U19s. The incidence during matches was 3.4 times higher (6.9 per 1000 h; 2.9–11.0 95% CI) than during training (2.0 per 1000 h; 1.5–2.2; 95% CI). The high incidence of injuries in U16 and U19 was mainly due to the high incidence of injuries during match play (13.9 per 1000 h of match play and 14.5 per 1000 h of match play, respectively). The season incidence rate of players with at least one injury was 66/138 = 47.8%. Descriptive injury outcomes by age are shown in Table 2.

**Figure 1:**
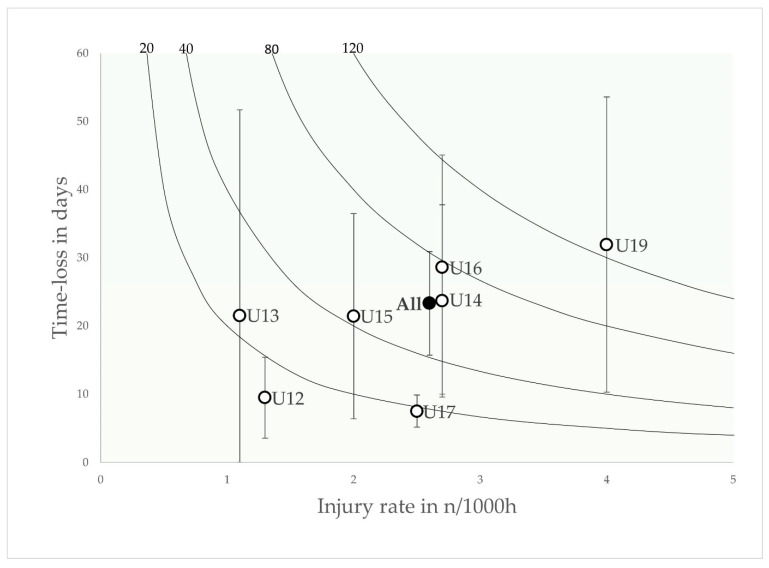
Risk matrix illustrating the age-related injury burden in elite youth footballers—injury burden is shown as the product of incidence and severity. Error bars indicate 95% CI. Isobars illustrate the injury burden in 5, 10, 20 and 30 days lost per year.

The total number of days lost was 2536, resulting in an injury burden of 60.6 days lost per 1000 h (40.8–80.3; 95% CI). The mean severity was 23.3 days per injury (15.7–30.9; 95% CI), with a higher severity in matches 28.9 (10–47.8 days; 95% CI) than in training 20.4 (13.7–27 days; 95% CI; *p* > 0.05). The injury burden for match injuries (199.4 days per 1000 h) was 4.9 times higher than for training injuries (40.8 days per 1000 h). A risk matrix shows the injury burden as a variable dependent on the incidence and severity of injury for each age group (Figure 1).

### 3.2. Injury Mechanism and Injury Type

In total, 86 of the 109 time-loss injuries were traumatic. Traumatic injuries accounted for 92% of match injuries and 72% of training injuries. Overuse injuries accounted for 21% of all injuries. However, the exact mechanisms of trauma were not recorded.

The most common site of injury was the lower extremity (83.4%; Table 3), followed by the upper extremity (9.2%), head and neck (4.6%), and trunk (2.8%).

The injury types with the highest incidence were joint capsule and ligament injuries (0.7; 30%), muscle and tendon injuries (0.7 injuries per 1000 h; 28% of all injuries), bone injuries (0.3; 11%) and superficial tissue/skin injuries (0.3; 11%). Bone injuries were associated with the highest injury burden (12.8, 7–19 days per 1000 h, 21% of all days lost), followed by joint sprains (10.1, 7–14 days per 1000 h, 20%), physeal injuries (9.2, 6–13 days per 1000 h, 15%), muscle injuries (8.6, 6–11 days per 1000 h, 14%) and cartilage lesions (6.2, 2–13 days per 1000 h, 10%).

### 3.3. Age Group Patterns

**Figure 2:**
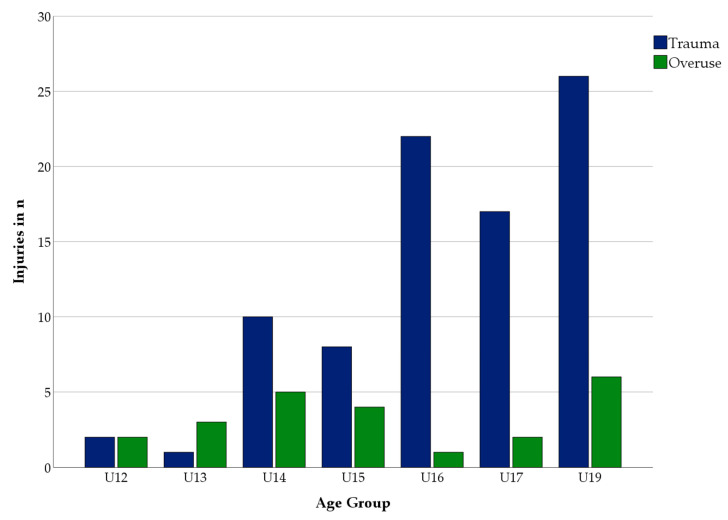
Number and proportion of overuse and traumatic injuries by age group.

The total number of overuse injuries and their proportion of all injuries in an age group decreased with increasing age (U12: 50%, U13: 75%, U14: 33%, U15: 33%, U16: 5%, U17: 12%, U19: 23%). In contrast, the number of traumatic injuries increased with age, as shown in Figure 2.

**Figure 3:**
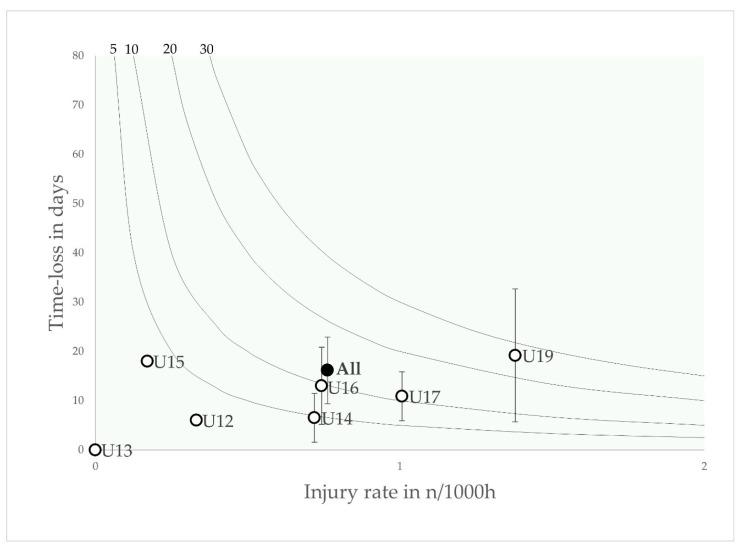
Risk matrix illustrating the age-related injury burden for joint sprains in elite youth footballers—injury burden is shown as the product of incidence and severity. Error bars indicate 95% CI. Isobars illustrate the injury burden in 5, 10, 20 and 30 days lost per year.

**Figure 4:**
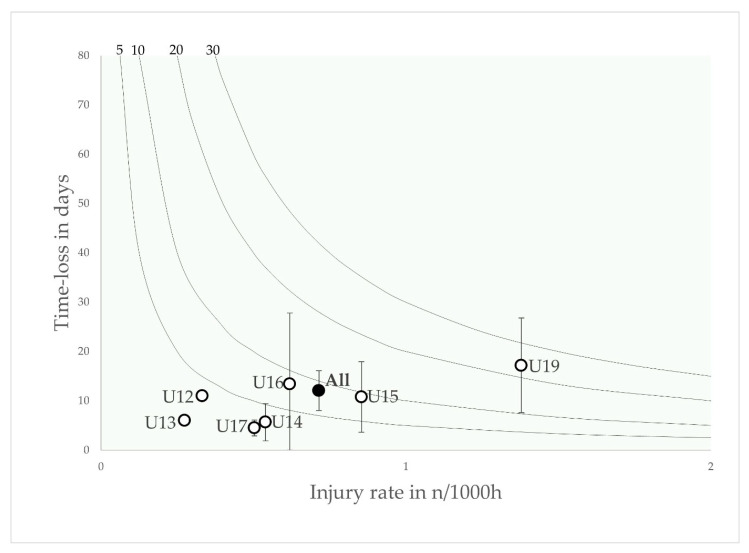
Risk matrix illustrating the age-related injury burden for muscular injuries in elite youth footballers—injury burden is shown as the product of incidence and severity. Error bars indicate 95% CI. Isobars illustrate the injury burden in 5, 10, 20 and 30 days lost per year.

**Figure 5:**
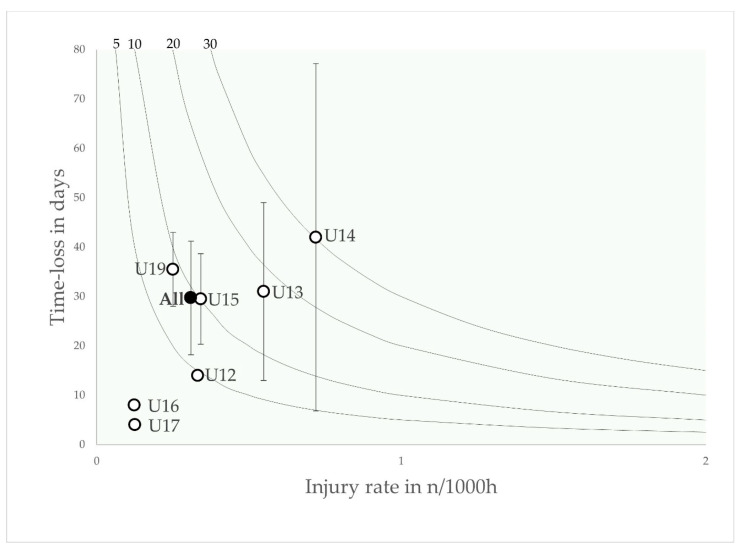
Risk matrix illustrating the age-related injury burden for physeal injuries in elite youth footballers—injury burden is shown as the product of incidence and severity. Error bars indicate 95% CI. Isobars illustrate the injury burden in 5, 10, 20 and 30 days lost per year.

**Figure 6:**
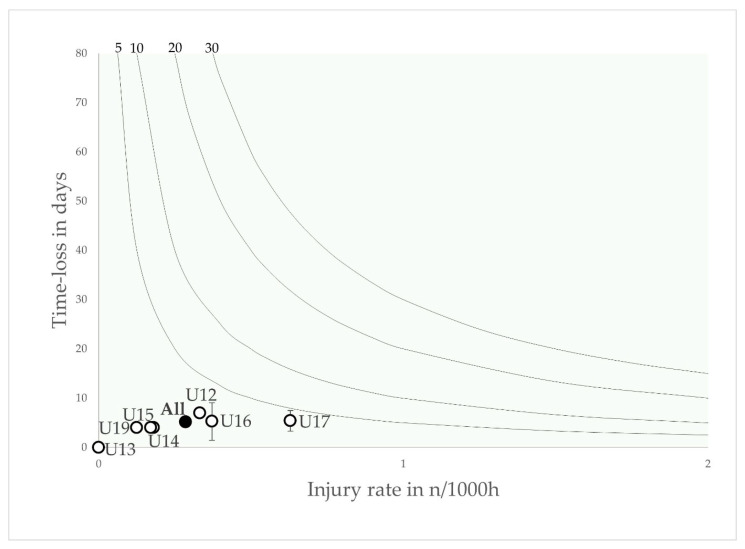
Risk matrix illustrating the age-related injury burden for contusions in elite youth footballers—injury burden is shown as the product of incidence and severity. Error bars indicate 95% CI. Isobars illustrate the injury burden in 5, 10, 20 and 30 days lost per year.

**Figure 7:**
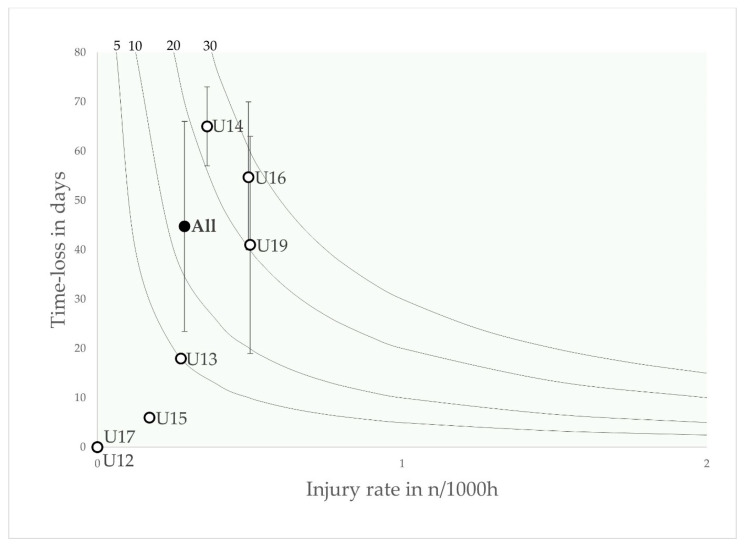
Risk matrix illustrating the age-related injury burden for bone injuries in elite youth footballers—injury burden is shown as the product of incidence and severity. Error bars indicate 95% CI. Isobars illustrate the injury burden in 5, 10, 20 and 30 days lost per year.

The incidence and severity of the five most burdensome injury types are shown as risk matrices in Figure 3, Figure 4, Figure 5, Figure 6 and Figure 7.

Injury burden, as a variable dependent on injury incidence and severity, is shown as 5, 10, 20 and 30 days lost per 1000 h, plotted in four steps with isobars. The highest incidence of joint sprains was found in the U16, U17 and U19 age groups, with the highest injury burden observed in the U19 age group.

Muscular injuries were most common in the U15, U16 and U19 age groups, with a peak injury burden in the U19 age group. They played a minor role in the U12, U13 and U14 age groups.

Physeal injuries occurred mainly in the U13, U14 and U15 age groups, with the highest injury burden in the U14 age group.

For contusions and fractures, there was no trend in the incidence and burden of injury between the different age groups. However, bone injuries were associated with a high injury burden.

## 4. Discussion

In the development of young athletes, the loss of time due to injuries leads to a lack of improvement in sport-specific technique, a deficit in physical development that is difficult to compensate for and, frequently, a lack of support from the association or club [23]. In football, injuries are often associated with significant financial risks. As injury characteristics vary between populations and age groups, it is essential to provide national insights.

Injury characteristics in elite German youth academies have recently been published [15]. The aim of this study was therefore to differentiate age-related differences in injury risk, injury severity and injury burden, and thus to draw conclusions about the age-related risk profile of 138 elite youth footballers.

### 4.1. Main Injury Outcomes

It is often argued in the literature that higher levels of endurance, strength and coordination may lead to lower injury rates [24]. Recently, however, studies from elite international youth football have been published that contradict this [12,16]. This hypothesis also cannot be confirmed in the present collective. On the contrary, the total incidence of injuries varied between 1.1 and 4.0 per 1000 h of exposure and increased gradually with age. This is at the lower end but comparable to the 4.3–5.8 per 1000 h incidence rates reported for adults in the UEFA Champions League or Swedish professional football [25,26]. It is possible that “playing up” (i.e., playing against older age groups) has led to an inversion of the injury rate in young athletes in the studies described [24]. While growth appears to have a large influence on physeal injuries, physical play and physical strength appear to have a greater influence on injury rates and burden in youth football [8]. Therefore, it is important that coaches, coaching staff, physiotherapists and physicians discuss potential risk factors related to the growth phase and physical competitiveness and, if necessary, design individual training and match plans to reduce the risk of injury in these players [13,27].

The balance between match and training load appears to be particularly important. In the present collective, a 3.4 times higher incidence rate and a 4.9 times higher injury burden rate were observed for match-associated injuries compared to training-associated injuries. This was due to the increased incidence and severity of training and match injuries, particularly in older age groups. The fact that the ratio of match/training related injuries increases with the age of the young athletes is known from other studies in elite football academies [12,28]. The incidence data from the academy studied were within the range of published incidence data from youth academies in other countries and there were no significant differences in training incidence of 0.7–7.9 per 1000 h and match incidence of 0.4–38.4 per 1000 h [11,24]. While some factors, such as athletic competition and increased physicality, are inherent to the nature of the game, it is important to ensure good physical condition and mental fitness, to minimise the risk of injury during the match [29].

Further comparison of injury data shows that the U19 and U16 age groups have the highest injury burden, with 128 and 88 days lost per 1000 h, respectively. This is a valuable finding as there are few and conflicting reports on the injury burden in elite youth football. While one study in a Dutch cohort found a peak injury burden of 357 days lost per squad season in the U16 age group, a cohort of Qatari and Belgian youth footballers showed an increasing injury burden with age [11,12,30]. Furthermore, the overall burden of 61 days lost per 1000 h of exposure found in the present study needs to be discussed. Due to different reporting of injury burden (number of days lost per 1000 h vs. number of days lost per squad season), comparability is rarely possible [11,12,16,30]. According to the consensus, the injury burden should be reported as number of days lost per 1000 h [21,22]. As exposure during a season varies between teams, leagues and countries, the specification per 1000 h of exposure is concrete. However, in some studies the exposure data of the athletes are missing [11,12,30]. As a result, a study from a Qatari youth academy is the only study currently available that reports exposure data and injury burden [16]. The reported overall burden rate is higher than in the current collective and is reported as 255 days of absence per 1000 h [16].

Overall, the injury burden in the risk matrix of the Qatari youth football collective is more homogeneous, whereas in the present study there is a large difference in the injury burden observed between younger (U12: 12.4 days lost per 1000 h and U13: 23.7 days lost per 1000 h) and older age groups (U16: 77.2 days lost per 1000 h and U19: 127.6 days lost per 1000 h) [16]. However, the data matrix of the present study (Figure 1) is comparable to those of previous studies [12]. A potential reason for these differences might be differences in exposure recording. In the present study, match exposure was recorded on an individual basis, whereas training exposure was assessed at team level. In contrast, the training and match exposures in the reference study by Wik et al. were assessed on an individual basis, which allows a more reliable assessment [16]. However, due to discrepancies between the textual reporting and the data presented in the risk matrices, the exact injury burden of the study mentioned by Wik et al. remains unclear [16]. A risk matrix showed just under 100 days lost per 1000 h, while the data reported in the text suggested 225 days lost per 1000 h [16].

Further studies with appropriate exposure assessment are needed to provide further reference values for injury burden, measured in days lost per 1000 h of exposure.

### 4.2. Onset Mechanism, Injury Type and Age Group Patterns

Musculoskeletal, joint capsular and ligamentous injuries were the most frequent and were associated with moderate severities. Muscle injuries accounted for 28% of all injuries and particularly affected the thigh muscles. In professional football, this type of injury was reported in 31% of all injuries [31]. Muscle injuries were associated with an injury burden of 8.6 days lost per 1000 h, with the frequency and severity of injuries being particularly high in the U19, U16 and U15 age groups. Thigh muscle injuries receive special attention in terms of prevention and treatment [32]. Muscle injuries with a particular focus on the thigh muscles, are a significant problem due to a complex interplay of factors [33]. These factors include growth-related imbalances, developmental differences in the musculature of young athletes, muscular rigidity, non-adherence to prevention programmes and sport-specific elements such as kicking and rapid changes of direction [34]. Efforts to reduce hamstring injuries have led to the development of the Nordic Hamstring Exercise Programme, which has shown remarkable efficacy in reducing such injuries [35]. However, the effectiveness of alternative training programmes, such as plyometric training, remains controversial in the literature [34]. Paradoxically, despite the existence of these effective preventive strategies, the incidence of hamstring injuries continues to increase in both professional and amateur football [31]. This concerning trend may be due to a lack of awareness of the effectiveness of these programmes and an unwillingness to participate in specific training [36]. An observed increase in physicality, measured by high-intensity sprints and actions, as well as sprint distance and number, are also seen as potential influencing factors [37]. Further research and therapeutic approaches are needed to counteract this trend.

Another finding of the present study was the reverse development of the incidence of overuse and traumatic injuries in the different age groups. In line with the increase in traumatic injuries, the injury burden of fractures, joint sprains and muscle injuries increased with age. In parallel with the incidence of injuries, the severity of injury types increased with age. Possible reasons for this could be a higher intensity of play with increasing age, a higher training load, a higher level of competition and a more aggressive and therefore riskier style of play [38]. The increasing incidence of traumatic injuries may also be due to an increased likelihood of previous injury prior to the study period, which is a known risk factor for new injuries in youth football [39].

Furthermore, the age-specific distribution of injury burden for specific injury types shows an interesting age-dependent distribution of peak injury burden, especially for physeal injuries. Physeal injuries occurred mainly during the phases of the pubertal growth spurt between U13 and U15, with a peak in the U14 age group, and were associated with high injury incidence and severity. The lower burden of physeal injuries in older age groups may be due to advanced skeletal maturation [40]. There is a further increase in the injury burden in the U19 age group, which can be interpreted as a correlate of the final growth of young males [11]. The apophyses are highly vulnerable regions of the body that are very sensitive to repetitive and excessive forces. The vulnerability of the apophyses is particularly increased during periods of rapid physical growth [41]. In the present cohort, apophyseal injuries accounted for 12% of all time-loss injuries and 29% of all serious injuries with a time-loss of more than 4 weeks. Comparable values of between 0.4 and 4.7 injuries per squad season can be found in the literature, but the injury burden in the present cohort is high and has not been extensively studied in the literature [7,8,9,10]. The high injury burden may be due to the increasing intensity of elite youth football, which leads to greater mechanical stress on the growth plates of the lower limbs [42]. However, when compared to the only previously published data on a Qatari cohort of young footballers, which reported a physeal burden of 33–49% of the total burden, or a range of 208 to 444 days lost per squad season, it is lower and, at 15% in the present cohort, has a smaller impact on the total burden [43]. Nevertheless, the proportion of the total burden is a cause for concern, as it appears to have a detrimental effect on player development and therefore underlines the need for effective prevention programmes.

Monitoring of growth and maturation, tailored training programmes and careful management of football exposure appear to be an effective approach to reducing the risk, and consequently the burden, of apophyseal and overuse injuries in the target age groups of elite youth football [44].

### 4.3. Strength and Limitations

There are some limitations that need to be mentioned. The recording of injuries by the heterogeneous and large staff of the academy studied is a likely source of bias, despite the prior training of the staff. Fewer people involved in the collection of exposure data could reduce this bias in future studies. It should also be noted that match exposure data were recorded individually, whereas training exposure data were recorded for the whole team. This leads to a potential overestimation of training exposure and a subsequent underestimation of injury incidence and burden. However, for operational and staffing reasons, it was not possible to collect more comprehensive exposure data in the academy studied. Future studies should consider this as a potential bias and, if possible, collect both match and training exposure data individually for each athlete.

Another limitation is that the present study only collects data from a single elite football academy in Germany, resulting in a lack of external validity. Such data could be collected, for example, by implementing registry structures, which are already used in other sports orthopaedic subspecialties [45]. However, assessing overall injury rates and differences between clubs may be limited by differences in training, coaching methods and match types. Furthermore, sharing such data may be challenging due to confidentiality issues between professional football clubs [24]. A more valuable approach may be longitudinal research over several seasons within a single academy, including position-specific injury analysis.

In this study, all academy and club activities during the season were recorded, but non-scheduled activities and exposure to the national team were not monitored. These factors may have influenced the incidence of injuries, particularly gradual onset injuries [5]. It is also important to note that contextual factors related to training philosophies, lifestyle habits and environmental conditions should be considered when applying the findings of this study.

This prospective cohort study of elite youth football players provides a detailed and novel insight into the age-related injury characteristics and injury burden in a German youth academy. This is an important contribution to the clinical and preventive aspects of injury research in elite athletes, as recently emphasised [21].

## 5. Conclusions

This study provides an insight into the age-related injury characteristics of young elite footballers in Germany. The players missed a total of 2536 days, resulting in an injury burden of 60.6 lost days per 1000 h of exposure (40.8–80.3; 95%-CI). The injury burden for match-related injuries was 4.9 times higher than for training, with 199.4 lost days per 1000 h. Bone injuries (12.8 days lost per 1000 h), joint sprains (10.1), physeal injuries (9.2), muscle injuries (8.6) and cartilage lesions (6.2) were associated with the highest injury burden. With increasing age, the incidence, severity and burden of injury increased significantly, which may be explained by the increasing number of traumatic injuries and may be due to the increased physicality of the game in these groups. Physeal injuries, mostly related to overuse, peaked in the U14 age group during the pubertal growth spurt.

By categorising players by age group, the study provides valuable insights to ensure the optimal development of young elite players by highlighting the age groups in which athletes are most susceptible to injury during training and competition.

## Figures and Tables

**Table 1:** Definitions used in the study.

Measure	Definition
Player season	A player who participated in a particular season.
Time-loss injury	A physical condition or symptom experienced by a player that requires the medical staff to restrict, in whole or in part, the player’s participation in a future football team training session or match [20].
Injury incidence	The frequency of time-loss injuries per 1000 player hours [20].
Season incidence proportion	Percentage of players with at least one time-loss injury recorded for the season studied [16].
Injury burden	A measure of the impact of injury as a product of frequency (how often) and severity (duration), calculated as the total number of days lost per 1000 player hours [21].
Training exposure	Team and individual physical activities under the control of the team’s coaching or fitness staff, designed to maintain or improve football skills or physical condition. Team exposure is measured in hours [20].
Match exposure	Match between teams from different clubs or academies. Player exposure is measured in hours [22].

**Table 2:** Demography, exposure and injury characteristics.

	U12	U13	U14	U15	U16	U17	U19
**Players (n)**	15	18	20	21	22	21	21
**Age (y, SD)**	11.8 (0.4)	12.7 (0.5)	13.5 (0.5)	14.6 (0.5)	15.7 (0.5)	16.6 (0.5)	18.0 (0.8)
**Stature** **(cm, SD)**	148.1 (7,1)	156.7 (7.2)	161.4 (7.0)	168.5 (7.3)	173.8 (6.4)	176.7 (5.2)	181.7 (7.8)
**Body mass (kg, SD)**	42.1 (5.5)	44.7 (6.1)	48.3 (5.9)	55.4 (6.1)	62.5 (6.2)	69.8 (6.7)	75.1 (7.5)
**Time-loss injuries (n)**	4	4	15	12	22	20	32
**Total time-loss (days)**	38	86	356	257	629	148	1022
**Overall Injury incidence**	1.3	1.1	2.7	2.0	2.7	2.5	4.0
**Training injury incidence**	1.3	1.1	2.1	2.2	1.8	1.8	2.7
**Match injury incidence**	1.5	1.2	6.6	1.2	13.9	9.2	14.5
**Mean days lost per injury (95%-CI)**	9.5 (3.6–15.4)	21.5 (0–51.7)	23.7 (9.6–37.8)	21.4 (6.4–36.5)	28.6 (10–45.1)	7.5 (5.2–9.9)	31.9 (10.3–53.6)
**Injury burden (95%-CI)**	12.4 (4.7–20)	23.7 (0–56.9)	64 (25.9–102.1)	42.8 (12.8–73)	77.2 (27–121.8)	18.8 (13–24.8)	127.6 (41.2–214.4)

**Table 3:** Data on injury incidence, severity and burden by body part and diagnosis.

Body Part	Injuries Incidence Rate		Median Time-Loss	Burden
Diagnosis	n	Injuries Per 1000 h	Days (IQ 25th–75th)	Time Loss Days Per 1000 h (95% CI)
**Head and Neck**	5	0.12	10 (4.5–12.5)	1.1 (0.4 to 1.7)
Concussion	2	0.05	10.5 (10–10.5)	0.5 (0.2 to 0.8)
Nasal bone fracture	2	0.05	9 (4–9)	0.5 (0.2 to 0.8)
Cut	1	0.2	5 (5–5)	1 (1 to 1)
**Upper Limb**	10	0.24	7 (4.75–31.75)	5.4 (0.2 to 11.0)
AC-contusion	1	0.02	4 (4–4)	0.1 (0.1 to 0.1)
Shoulder contusion	1	0.02	5 (5–5)	0.1 (0.1 to 0.1)
Ellbow contusion	1	0.02	8 (8–8)	0.2 (0.2 to 0.2)
Forearm contusion	1	0.02	7 (7–7)	0.1 (0.1 to 0.1)
Forearm fracture	2	0.05	45.5 (45–46)	2.3 (1.4 to 3.7)
Hand contusion	2	0.05	5.5 (5–6)	0.3 (0.2 to 1.2)
Hand/finger sprain	1	0.02	6 (6–6)	0.1 (0.1 to 0.1)
Hand/finger fracture	1	0.02	93 (93–93)	1.9 (1.9 to 1.9)
**Trunk**	3	0.07	11 (5.0–11)	1.0 (0.4 to 1.8)
Overuse unspecific pathology	2	0.05	18.5 (11–18.5)	0.9 (0.6 to 1.3)
Functional muscle disorder	1	0.02	5 (5–5)	0.1 (0.1 to 0.1)
**Hip and Pelvis**	5	0.12	36 (13.5–58.5)	4.3 (1.0 to 7.7)
Physeal injury (avulsion)	2	0.05	34 (12–34)	1.7 (0.6 to 2.8)
Physeal injury (apophysitis)	2	0.05	48.5 (36–48.5)	2.4 (1.8 to 3.1)
Gluteal strain	1	0.02	15 (15–15)	0.3 (0.3 to 0.3)
**Thigh**	26	0.62	6.0 (4–32.5)	7.6 (4.6 to 10.5)
Torn hamstrings	5	0.12	23.0 (13–44)	3.4 (1.1 to 5.6)
Torn quadriceps	1	0.02	15 (15–15)	0.3 (0.3 to 0.3)
Thigh contusion	3	0.07	5 (4–5)	0.4 (0.2 to 0.5)
Adductor strain	5	0.12	11 (5.0–17.5)	1.3 (0.2 to 2.5)
Hamstring strain	4	0.1	4 (4–4.75)	0.4 (0.3 to 0.5)
Quadriceps strain	8	0.19	6 (4–13)	1.8 (0.4 to 3.1)
**Knee**	21	0.5	14 (7.5–38)	22.1 (4.2 to 40)
Knee sprain/ligament	9	0.21	10 (4–16)	2.4 (1.2 to 3.6)
Meniscus and cartilage damaged	3	0.07	48 (33–48)	6.2 (2.9 to 9.5)
Knee physeal injury	7	0.17	14 (8–28)	3.2 (1.1 to 5.3)
Patellar dislocation	1	0.02	90 (90–90)	1.8 (1.8 to 1.8)
Torn ACL	1	0.02	339 (339–339)	6.8 (6.8 to 6.8)
**Lower Leg and Calf**	6	0.14	7.5 (5.75–9.75)	1.1 (0.7 to 1.5)
Functional muscle disorder	1	0.02	12 (12–12)	0.2 (0.2 to 0.2)
Torn calf muscle	1	0.02	8 (8–8)	0.2 (0.2 to 0.2)
Calf muscle strain	3	0.07	6 (5–6)	0.5 (0.1 to 0.8)
Calf cut	1	0.02	7 (7–7)	0.1 (0.1 to 0.1)
**Foot and Ankle**	33	0.79	14 (5–31)	18.0 (11.3 to 24.7)
Ankle sprain/ligament	19	0.45	13.0 (7–19)	7.0 (3.5 to 10.4)
Ankle contusion	1	0.02	4 (4–4)	0.1 (0.1 to 0.1)
Ankle fracture	1	0.02	57 (57–57)	1.1 (1.1 to 1.1)
Ankle stress injury	1	0.02	5 (5–5)	0.1 (0.1 to 0.1)
Physeal injury	2	0.05	44 (39–44)	2.2 (2.0 to 2.5)
Foot/toe fracture	4	0.1	56 (17–65.8)	4.6 (0.2 to 9.0)
Foot contusion	3	0.07	4 (4–4)	2.2 (0.3 to 4.2)
Foot sprain	2	0.05	11 (4–11)	0.6 (0.2 to 0.9)

## Data Availability

The data presented in this study are available on request from the corresponding author. The disclosure of sensitive data of professional footballers may be prohibited by the participating club.
